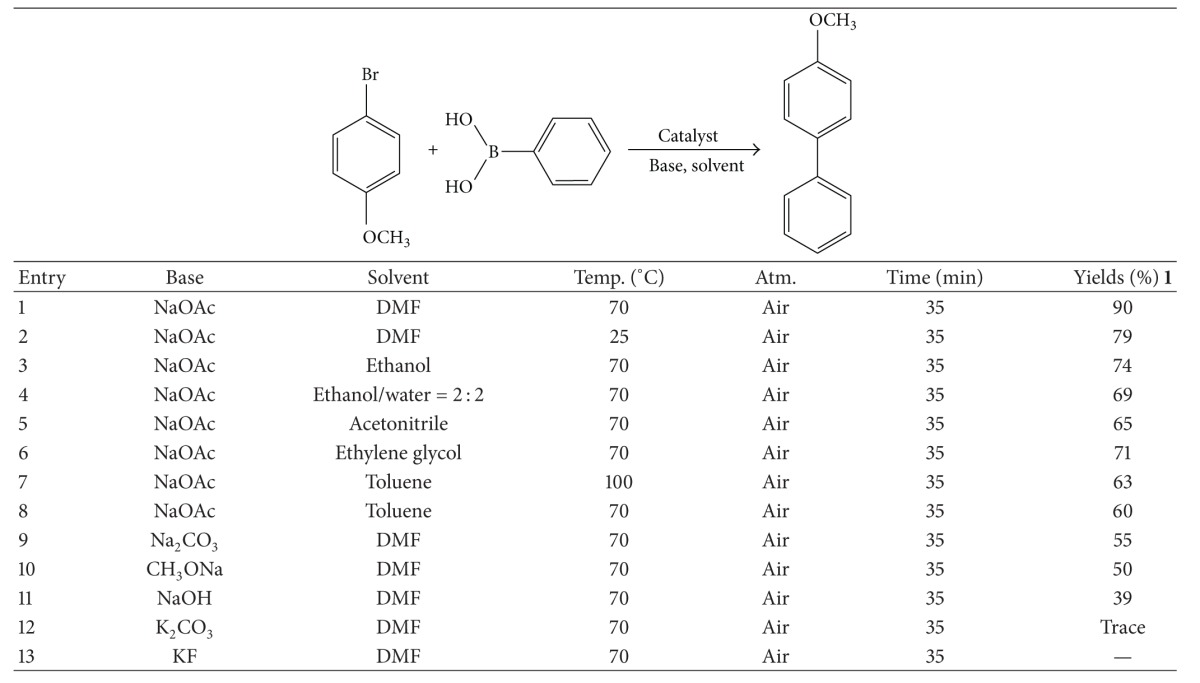# Pd(II) and Zn(II) Based Complexes with Schiff Base Ligands: Synthesis, Characterization, Luminescence, and Antibacterial and Catalytic Activities

**DOI:** 10.1155/2013/956840

**Published:** 2013-11-06

**Authors:** Zhi-Qiang Feng, Xiao-Li Yang, Yuan-Feng Ye

**Affiliations:** School of Material Engineering, Jinling Institute of Technology, Nanjing 211169, China

## Abstract

Two new metal complexes involving Schiff base ligands, namely, [Pd(L1)_2_] (**1**) and [Zn(L2)_2_] (**2**), [**HL1**: 2,4-dibromo-6-((E)-(mesitylimino)methyl)phenol and **HL2**: 2-((E)-(2,6-diisopropylphenylimino)methyl)-4,6-dibromophenol], have been solvothermally synthesized and characterized by elemental analysis, IR-spectroscopy, thermogravimetric analysis, powder X-ray diffraction, and single-crystal X-ray diffraction. Both **1** and **2** are mononuclear cyclometalated complexes with square planar and tetrahedral coordination geometry, respectively. **1** and **2** display photoluminescence in the solid state at 298 K (fluorescence lifetimes **τ** = 5.521 **μ**s at 508 nm for **1**; **τ** = 3.697 **μ**s at 506 nm for **2**). These Schiff base ligands and their metal complexes have been screened for antibacterial activity against several bacteria strains, and the results are compared with the activity of penicillin. Moreover, the Suzuki reaction of 4-bromoanisole with phenylboronic acid by **1** has also been studied.

## 1. Introduction

It is wellknown that Schiff bases are important compounds because of their wide range of biological activities [1] and as being ligands in conjunction with transition metals. These transition metals based compounds display diverse structural features and in some instances exhibit interesting reactivities, bacteriostasis, and photoluminescence [2–6]. Compared to platinum(II) complexes involving Schiff base ligands, whose applications as luminescent sensors are of considerable interest in inorganic photochemistry [7, 8], relatively scant attention has been focused upon the luminescent characteristics of palladium(II) complexes [9]. Meanwhile, the palladium catalyzed formation of biaryls from aryl halides with arylboronic acids (the Suzuki reaction) has become a mainstay of modern synthetic organic chemistry for the formation of carbon-carbon bonds [10, 11]. Moreover, Zn(II) complexes bearing salicylaldiminato ligands have been employed as blue, greenish-white, and red emitters in organic optoelectronics with better stability and efficiency [12, 13]. In those zinc(II) complexes, the salicylaldiminato ligands are mainly salen ligands with two N and two O donor atoms. However, reports on the luminescent properties of bis(salicylaldiminato) zinc(II) complexes are still limited. On the basis of the above considerations, we here report the synthesis, characterization, luminescence, and antibacterial and catalytic activities of two new transition metal complexes based on Schiff base ligands. 

## 2. Experimental

### 2.1. Materials and Methods

All chemicals were commercially available and used as received without further purification. 

### 2.2. Physical Measurements

Elemental analyses for C, H, and N were carried out using a Vario EL III Elemental Analyzer. Infrared spectra were recorded (4000–400 cm^−1^) as KBr disks on a Shimadzu IR-440 spectrometer. Thermogravimetric analyses (TGA) were performed on an automatic simultaneous thermal analyzer (DTG-60, Shimadzu) under a flow of N_2_ at a heating rate of 10°C/min between ambient temperature and 800°C. Powder XRD investigations were carried out on a Bruker AXS D8-Advanced diffractometer at 40 kV and 40 mA with Cu-K*α* (*λ* = 1.5406 Å) radiation. Luminescence spectra and lifetimes for crystalline samples were recorded at room temperature on an Edinburgh FLS920 phosphorimeter. Nuclear Magnetic Resonance spectra were recorded on a Bruker Avance 400 MHz spectrometer. ^1^H-NMR chemical shifts are reported in ppm from tetramethylsilane with the solvent resonance as the internal standard (CDCl_3_, *δ* = 7.26). Data are reported as follows: chemical shift (*δ* ppm), multiplicity (s = singlet, d = doublet, t = triplet, and m = multiplet), coupling constants (Hz), integration, and assignment. ^13^C-NMR spectra were collected on a 100 MHz spectrometer with complete proton decoupling. Chemical shifts were reported in ppm from the tetramethylsilane (TMS) with the solvent resonance as internal standard (CDCl_3_, *δ* = 77.23). Melting points (uncorrected) were measured with a Mel-Temp apparatus.

## 3. Solvothermal Synthesis

### 3.1. Preparation of **HL1**

**Scheme 1:**
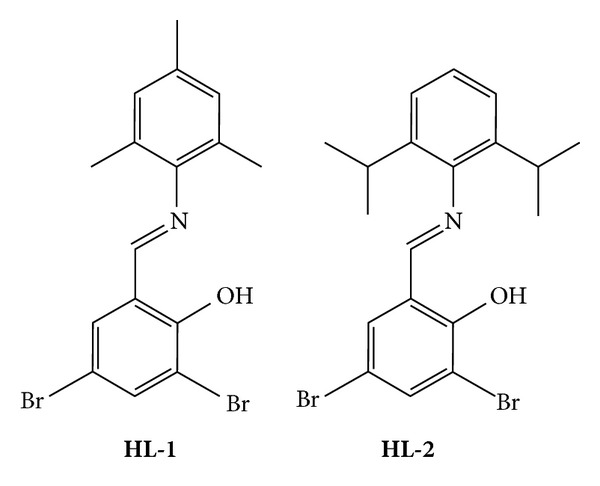
The molecular structures of **HL1** and **HL2**.

The preparation of **HL1** was carried out according to the reported procedures [14]. Yield 6.013 g (72%). ^1^H NMR (400 MHz, CDCl_3_) *δ*: 14.32 (s, 1H, O**H**), 8.20 (s, 1H, C**H**N), 7.24–7.77 (m, 4H, Ar–**H**), 2.22 (d, *J* = 51.2 Hz, 9H, C**H**_3_); ^13^C NMR (100 MHz, CDCl_3_) *δ*: 164.6, 157.9, 144.4, 138.4, 135.7, 133.2, 129.5, 128.7, 120.4, 112.5, 110.3, 20.7, 18.3; MP: 90–93°C. FTIR (KBr, cm^−1^): 3462(s), 2920(m), 2891(m), 1628(s), 1560(w), 1490(m), 1449(m), 1374(w), 1295(w), 1266(w), 1224(w), 1201(w), 1198(w), 1155(w), 1134(w), 1125(m), 1034(m), 992(w), 877(m), 849(m), 801(w), 765(w), 724(w), 688(s), 551(w), 485(w), 442(w), as shown in Scheme 1.

### 3.2. Preparation of **HL2**

**HL2** was prepared by the same procedure as for **HL1** except that 2,4,6-trimethylaniline was replaced by 2,6-diisopropylaniline. Yield 6.11 g (71%). ^1^H NMR (400 MHz, CDCl_3_) *δ*: 14.11 (s, 1H, O**H**), 8.21 (s, 1H, C**H**N), 7.44–7.79 (m, 5H, Ar–**H**), 2.93 (d, *J* = 4.6 Hz, 12H, C**H**_3_); 1.18 (m, *J* = 4.6 Hz, 2H, C**H**(CH_3_)_2_). ^13^C NMR (100 MHz, CDCl_3_) *δ*: 164.9, 157.4, 144.9, 138.9, 138.3, 133.5, 126.2, 123.4, 120.2, 112.3, 110.3, 28.2, 23.6; MP: 135–137°C. FTIR (KBr, cm^−1^): 3460(s), 2958(s), 2915(w), 1612(vs), 1442(s), 1385(w), 1350(m), 1319(w), 1295(m), 1214(w), 1157(w), 1114(w), 1087(w), 1034(m), 992(w), 932(m), 859(m), 801(w), 765(w), 724(w), 688(s), 551(w), 512(w), as shown in Scheme 1.

### 3.3. Preparation of [Pd(L1)_2_] **(1)**

Pd(C_2_H_3_O_2_)_2_ (0.112 g, 0.5 mmol) was dissolved in methanol (15 mL). **HL1** (0.359 g, 1 mmol) was added, and the mixture was stirred at room temperature for 2 h under an anhydrous atmosphere. The resulting mixture was filtered under reduced pressure. The collected solid was washed with diethyl ether and dried in air to give yellow crystals that were purified by recrystallization from methylene chloride (10 mL) and hexane (10 mL). Yield 0.315 g (70%). Anal. For C_32_H_28_Br_4_N_2_O_2_Pd (%): Calcd. C 42.7, H 3.1, N 3.1; found C 42.2, H 3.5, N 3.7. FTIR (KBr, cm^−1^): 3458(s), 2960(m), 2921(w), 1604(vs), 1580(s), 1508(vs), 1462(s), 1450(m), 1384(w), 1361(w), 1327(w), 1298(w), 1253(w), 1220(w), 1178(w), 1112(m), 1058(w), 1032(w), 929(w), 918(w), 877(m), 815(m), 797(m), 754(s), 703(w), 669(w), 577(w), 505(w) 482(w), 435(w), 413(w).

### 3.4. Preparation of [Zn(L2)_2_] **(2)**

Complex **2** was prepared by the same procedure as **1** except that **HL1** was replaced with **HL2**. Yield 0.12 g (86%). Anal. For C_32_H_28_N_2_O_2_Br_4_Zn (%): Calcd. C 44.8, H 3.3, N 3.3; found C 45.3, H 3.2, N 3.0. FTIR (KBr, cm^−1^): 3465(s), 2975(m), 2916(s), 1603(vs), 1473(s), 1440(s), 1309(m), 1199(m), 1120(w), 1016(w), 997(w), 960(w), 945(w), 844(m), 756(s), 694(s), 623(w), 590(w), 555(m), 498(s), 476(m), 424(w), 401(s).

### 3.5. Antibacterial Activity Tests

*In vitro* bacterial activities of the Schiff base ligands and their metal complexes were tested using the paper disc diffusion method. The chosen strains were G(+) *S. aureus* and *B. cereus* and *Rhizopus* and *E. coli*. The liquid medium containing the bacterial subcultures was autoclaved for 20 min at 15 lb pressure before inoculation. The bacteria were cultured for 24 h at 35°C in an incubator. Mueller Hinton broth was used for preparing basal media for the bioassay of the organisms. Nutrient agar was poured onto a Petri plate and allowed to solidify. The test compounds were dissolved in DMF and added dropwise to 10 mm diameter paper discs placed in the center of the agar plates. The plates were then kept at 5°C for 1 h and transferred to an incubator maintained at 35°C. The width of the growth inhibition zone around the disc was measured after 24 h of incubation. Four replicates were taken for each treatment. In order to clarify any participating role of DMF in the biological screening, separate control studies were carried out with the solutions of DMF alone, and they showed no activity against any bacterial strains.

### 3.6. Catalytic Reactions

A mixture of 4-bromotoluene (1.0 mmol), phenylboronic acid (1.2 mmol), organic solvents (6 mL), bases (2.0 mmol), and 0.5 mol% of catalyst was stirred at 80°C under air. After the reaction, the catalyst was separated by filtration. The filtrate was dried over Na_2_SO_4_ and filtered. The products were quantified by GC-MS analysis (Shimadzu GCMS-QP5050A equipped with a 0.25 mm × 30 m DB-WAX capillary column). The typical GC-MS analysis program was as follows: set initial column temperature as 100°C, hold 2 min, ramp temperature to 280°C at 15°C/min, and hold for 5 min.

### 3.7. X-Ray Crystallography

Single crystal X-ray diffraction analyses of complexes **1**-**2** were performed on a Bruker Apex II CCD diffractometer operating at 50 kV and 30 mA using MoK*α* radiation (*λ* = 0.71073 Å). Data collection and reduction were performed using the APEX II software [15]. Multiscan absorption corrections were applied for all the data sets using SADABS, as included in the APEX II program [15]. The structure was solved by direct methods and refined by least squares on F^2^ using the SHELXTL program package [16]. All nonhydrogen atoms were refined with anisotropic displacement parameters. Hydrogen atoms attached to carbon and oxygen were placed in geometrically idealized positions and refined using a riding model. Crystallographic data are listed in Table 1. Selected bond lengths and angles and H-bonding parameters for compound **2** are given in Tables 2 and 3, respectively.

## 4. Results and Discussion 

### 4.1. Structure of **1**

**Figure 1:**
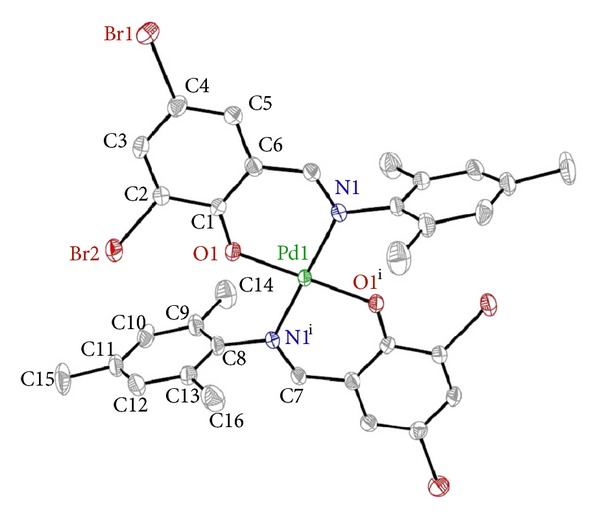
The molecular structure of compound **1 **with numbering scheme (30% probability ellipsoids). All H atoms are omitted for clarity. Symmetry code: (*i*) 2 − *x*, 1 − *y*, 2 − *z*.

Complex **1** crystallizes in the monoclinic space group* P*2_1_/*n* and contains one Pd(II) ion and two crystallographically independent **L1** ligands, as shown in Figure 1. In this complex, each Schiff base ligand (**L1**) is bonded to the Pd(II) center through nitrogen atom and oxygen atom, providing two equivalent six-membered N–Pd–O–C-chelate rings. The geometry at the Pd(II) center in **1** is square planar, with the two cyclometalated ligands in a *trans* arrangement. The Pd–O and Pd–N distances are 1.969(3) and 2.026(4) Å, similar to those seen in related complexes [4, 17]. In the six-membered chelate rings, the six atoms (Pd1, O1, C1, C6, C7, N1) are essentially planar. The planes of the two phenyl rings are inclined by 4.12(2) and 87.87(3)° to the PdN_2_O_2_ coordination plane, respectively. The bite angle [N1–Pd1–O1 = 93.04(4)°] is in good agreement with those found in a structurally related mononuclear complex with salicylaldiminato ligands as bridging ligands [12, 13]. 

### 4.2. Structure of **2**

**Figure 2:**
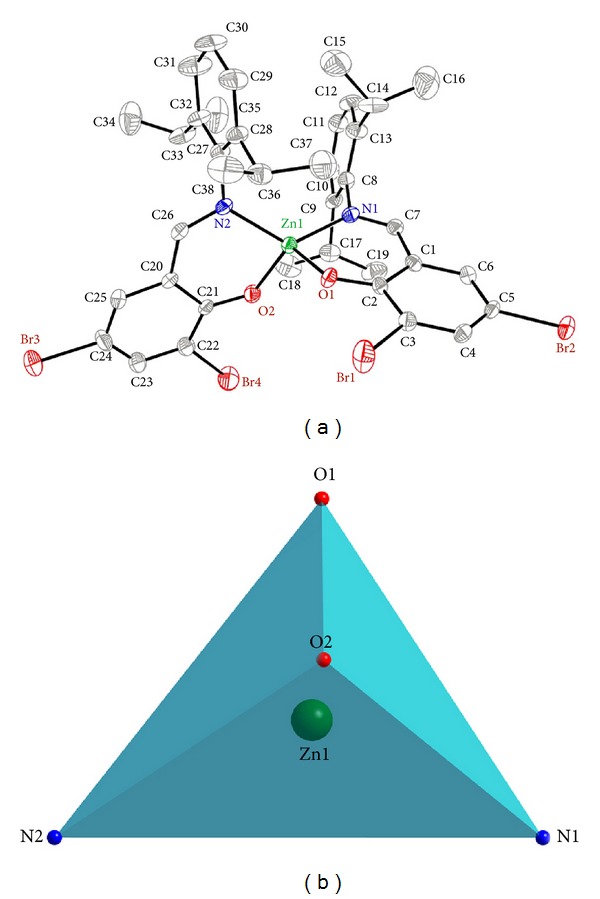
(a) The molecular structure of **2 **with numbering scheme (30% probability ellipsoids). All H atoms are omitted for clarity; (b) tetrahedral geometry surrounding the Zn(II) atom of **2**.

**Figure 3:**
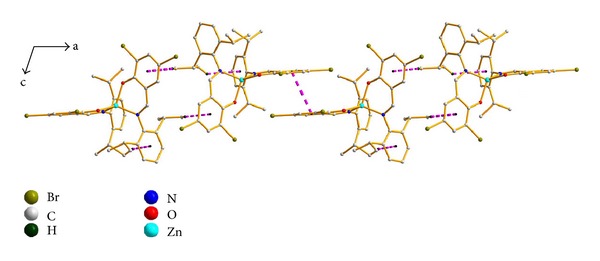
View of an infinite chain of **2** formed by intermolecular *π* ⋯ *π* and C–H ⋯ *π* stacking interactions with *pink dashes*.

X-ray crystallography shows that complex **2** crystallizes in the monoclinic space group *C*2/*c* and adopts a distorted tetrahedral geometry with the Zn^II^ center chelated by two bidentate **L2** ligands through the phenolate oxygen atom and imine nitrogen atom (Figures 2(a) and 2(b)). The O–Zn–O angle in **2** (111.32(16)°) is close to those values (105–112.5°) in the similar bissalicylaldiminato zinc complexes (NR^2^C_7_H_5-*x*_(R^1^)_*x*_O)_2_Zn [*x* = 1 or 2; R^1^ = Me, ^**t**^Bu, Cl, OMe; R^2^ = 2,6-^**i**^Pr_2_C_6_H_3_] [18], while the N–Zn–N angle in **2** (126.72(18)°) is also close to those in the latter complexes (122.9–128.9°). The two dihedral angles between the phenyl rings (C8–C13 and C27–C32) and the six-membered chelating ring (O1–Zn1–N1–C7–C1–C2) are 84.90 and 86.35°, respectively. The two six-membered chelating rings are nearly planar with the zinc atoms lying 0.513 and 0.310 Å out of the plane, and they are closely perpendicular to each other with the dihedral angle being 86.50°. The imino C=N bonds in **2** retain their double bond character, being 1.298(6) Å (C26=N2) and 1.256(6) Å (C7=N1) in length. The mononuclear molecules are connected into a 1D infinite chain through intermolecular *π* ⋯ *π* and C34–H34A ⋯ *π* stacking interactions between benzene rings (Cg1) of **L2** ligands, with a centroid-to-centroid distance of 3.786(7) Å (Figure 3) (Cg1 is the centroid of the C1–C6 ring). Intramolecular C15–H15B ⋯ *π* and C35–H35A ⋯ *π* stacking interactions further stabilize the 1D chain. The H-to-centroid distances of H34A ⋯ Cg2^*i*^ = 2.93(2) Å, H15B ⋯ Cg3 = 2.69(3) Å, and H35A ⋯ Cg4 = 2.93(7) Å, [Cg2, Cg3, and Cg4 are the centroid of the C20–C25, C27–C32, and C8–C13 rings, respectively, symmetry code: *i* = -*x*, *y*, 0.5-*z*]. Moreover, weak intramolecular C–H ⋯ N hydrogen bonds are also observed (Table 3).

### 4.3. Powder X-Ray Diffraction Analysis

**Figure 4:**
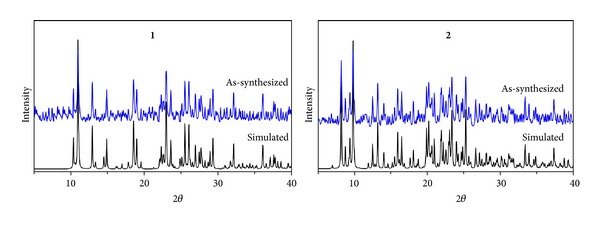
PXRD patterns in complexes **1**-**2**.

In order to check the purity of complexes **1**-**2**, bulk samples were measured by X-ray powder diffraction at room temperature, as shown in Figure 4. Although the experimental patterns have a few un-indexed diffraction lines and some are lightly broadened in comparison with those simulated from the single-crystal data using Mercury, it still can be well considered that the bulk synthesized materials and the crystals used for diffraction are homogeneous.

### 4.4. Infrared Spectra

FT-IR spectra of **HL1**, **HL2**, **1,** and **2** were recorded as KBr pellets (see Figure S1 in Supplementary Material available online at http://dx.doi.org/10.1155/2013/956840). In the IR spectrum, moderate bands at 2920 and 2891 cm^−1^ for **HL1**, 2958 and 2915 cm^−1^ for **HL2**, 2960 and 2921 cm^−1^ for **1,** and 2975 and 2916 cm^−1^ for **2** are associated with the methyl (–CH_3_) or methine (–CH–) stretching vibrations. The features at 1628 cm^−1^ for **HL1**, 1612 cm^−1^ for **HL2**, 1604 cm^−1^ for **1**, and 1603 cm^−1^ for **2** may be assigned to the –CH=N– stretching vibrations. The absorption peaks of –CH=N– group in **1** and **2** are clearly blue shifted against the related ligands, perhaps due to the conjugative effect between metal ions and ligands.

### 4.5. Thermogravimetric Analysis of **1**-**2**

**Figure 5:**
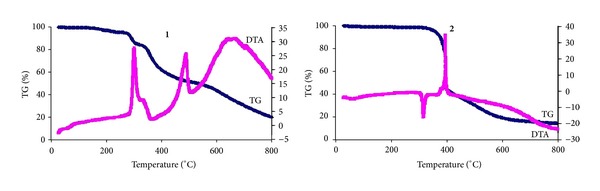
Thermogravimetric curves (TG and DTA) for complexes **1**-**2**.

The TG and DTA curves of **1**-**2** are shown in Figure 5. Both structures show good thermal stability as no clean weight-loss step occurs below 280°C for **1** and 320°C for **2**. The DTA trace of **2** shows superior endotherm to **1** indicating that **2** has better thermal stability than **1**. The weight-loss step above 280°C and 320°C corresponds to decomposition of the framework structures. Finally, **1** and **2** were completely degraded into PdO and ZnO, respectively, with total loss of 84.1 wt% (calcd. 86.4 wt%) for **1** and 89.7 wt% (calcd. 91.3 wt%) for **2**. 

### 4.6. Luminescent Properties

**Figure 6:**
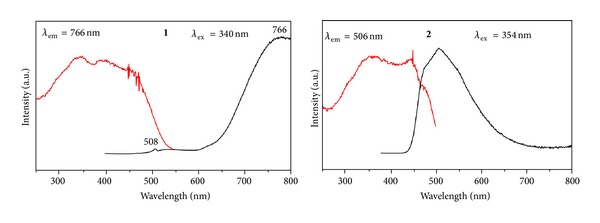
Solid-state excitation and emission spectra of **1 **and **2** at room temperature.

As part of a continuing program dedicated to luminescent transition systems, the spectroscopic behavior of complexes **1**-**2** is presented. The solid-state emission luminescence spectra of **1** at room temperature upon excitation at 340 nm are shown in Figure 6. The emission spectrum shows two bands at *λ*_max⁡_ = 508 and 766 nm, respectively. The solid-state excitation spectra for **1** reveal the presence of low-energy ligand-field states in the 300–400 nm spectral region. The high-energy structured band of **1** is assigned to a transition to ^3^IL excited state [19]. Complex **2** emits strong blue light in the solid state at room temperature with maximal emission wavelength at 506 nm (excitation wavelength 354 nm, Figure 6).

**Figure 7:**
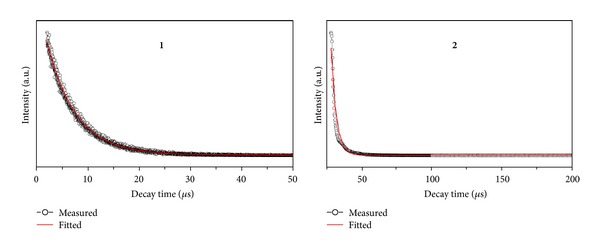
Luminescence lifetimes for complexes **1**-**2** in solid state at room temperature.

The coordinated zinc atom plays a dual role in the luminescence of **2** as pointed out in the literature for coordination complexes [20]. First, the formation of covalent bonds between the Zn and O atoms *viaπ*-donation of a lone-pair electrons on the O atom to the Zn atom changes the emission energy due to the lowering of the energy gap between π* and *π*. Second, the coordination of the ligands with the Zn atom increases the rigidity of the ligands, which can diminish the loss of energy *via* vibrational motions and increase the emission efficiency. Compared to the free ligands, the emission maxiumu of **1**-**2** is obviously red-shifted (see Figure S2) in the solid state, which may be assigned as metal-to-ligand charge transfer [21]. The luminescence lifetimes of solids 1-2 using an Edinburgh FLS S920 phosphorimeter with a 450 W xenon lamp as excitation source show lifetimes for 1 of *τ* = 5.521 *μ*s at 508 nm and for 2 of *τ* = 4.023 *μ*s at 506 nm (Figure 7). It should be noted that complexes **1** and **2** possess longer fluorescence lifetime than other palladium and zinc complexes involving Schiff bases [2, 22] and so could be used for light-emitting luminescent materials.

### 4.7. Biological Activity Tests

The susceptibility of certain strains of bacterium towards the ligands **HL1-HL2** and their metal complexes **1-2** was judged by measuring the size of their bactericidal diameters (*vide supra*). The results are given in Table 4. The effect against *Staphylococcus aureus* of ligands and complexes was found to be close to that of sodium penicillinate. All of the ligands and complexes showed inhibition diameters larger than sodium penicillinate against *Bacillus cereus*. Compared to **HL1-HL2** and **1-2** which show good activities, the penicillin is ineffective against *Escherichia coli*. However, similar to sodium penicillinate, the four tested samples showed no appreciable activity. Moreover, we found that the complexes were more effective than the ligands. It is possible that the ligand may be activated by the metal ion [23]. El-Sherif reported a series of palladium complexes that show antibacterial activities against *S. pyogenes* and *E. coli* bacteria at different concentrations 1, 2.5, and 5 mg/mL in DMSO in which the activities were smaller in the complexes than in ligands [24]. Also the results obtained for **1** and **HL** show better antibacterial activities compared to the platinum(II) and palladium(II) complexes based on Schiff bases [20].

### 4.8. Suzuki Reaction Catalysis of **1**

Pd(II) based complexes are well known for their catalytic activities [2, 5]. The Suzuki reaction of 4-bromoanisole with phenylboronic acid by **1** is reported here. The results reveal that the base and solvent for the Suzuki reaction greatly influence catalytic activity (Table 5). The reaction temperature has lower effect on the catalytic activity of **1**. Among six different organic solvents, DMF is found to the best solvent for this catalytic system. In other organic solvents, for example, ethanol, ethanol/water (2 : 2), ethylene glycol, acetonitrile, and toluene, relatively low yields of coupling products were obtained. Among five different bases investigated for these reactions, NaOAc was found to be the most effective (Table 5, entry 1); K_2_CO_3_, Na_2_CO_3_, CH_3_ONa, and NaOH were substantially less effective. KF failed to promote the reaction (Table 5, entry 13). Compared with other Pd(II) complexes based on Schiff base ligands, the catalytic activities of the present complexes for the Suzuki reaction proved to be highly effective [25].

## 5. Conclusion 

In summary, two new transition metal complexes based on Schiff base ligands have been successfully synthesized under solvothermal conditions. Both **1** and **2** show good thermal stability and exhibit photoluminescence in the solid state at room temperature (*τ* = 5.521 *μ*s at 508 nm for **1**, *τ* = 3.697 *μ*s at 506 nm for **2**), suggesting utility as light-emitting luminescent materials. The antibacterial activity tests showed that the ligands and complexes exhibited superior biological activity against *Staphylococcus aureus*, *Bacillus cereus*, and *Escherichia coli.* Moreover, complex **1** displays highly catalytic activities in the Suzuki coupling reaction of 4-bromoanisole with phenylboronic acid, which are also very sensitive to the choice of base and solvent.

## Supplementary Material

Figure: S1 The IR spectra of **HL1, 1 and HL2, 2**.
Figure: S2 Solid-state excitation and emission spectra of **HL1** and **HL2** at room temperature.

## Figures and Tables

**Table 1:** Crystal and refinement data.

Compounds	**1**	**2**
Empirical formula	C_32_H_28_Br_4_N_2_O_2_Pd	C_38_H_40_Br_4_N_2_O_2_Zn
Formula weight	898.60	941.73
Temperature (K)	296 (2)	296 (2)
Crystal system	Monoclinic	Monoclinic
Space group	*P*2_1_/*n*	*C*2/*c*
*a* (Å)	10.9718 (16)	20.161 (3)
*b* (Å)	11.8932 (17)	17.970 (3)
*c* (Å)	12.2321 (18)	23.087 (3)
*β* (°)	92.733 (3)	111.306 (3)
*V* (Å^3^)	1594.3 (4)	7793 (2)
*Z*, *D* (Mg·m^3^)	2, 1.872	8, 1.605
Limiting indices	−13 ≤ h ≤ 12, −14 ≤ k ≤ 13, −14 ≤ l ≤ 14	−24 ≤ h ≤ 24, −21 ≤ k ≤ 17, −27 ≤ l ≤ 26
Reflections collected/unique	8635/2853	21523/7002
*F*(000)	872	3744
*θ* (°)	2.39–25.20	1.57–25.20
Goodness-of-fit on *F* ^2^	1.098	0.984
*R* (*I* > 2*σ*)	*R*_1_ = 0.0345 *wR*_2_ = 0.1068	*R*_1_ = 0.0459 *wR*_2_ = 0.1267
*R* (all data)	*R*_1_ = 0.0542 *wR*_2_ = 0.1345	*R*_1_ = 0.0831 *wR*_2_ = 0.1565
Largest diff. peak and hole (Å^−3^)	0.936, −0.761	1.047 and −1.072

*R* = ∑(||*F*_*o*_|−|*F*_*c*_||)/∑|*F*_*o*_|.

*wR* = [∑*w*(*F*_*o*_^2^−*F*_*c*_^2^)^2^/∑*w*(*F*_*o*_)^2^]^1/2^.

**Table 2:** Selected bond length and angles of **1**-**2**.

Compound **1**			

Pd1–N1	2.026 (4)	Pd1–O1	1.970 (3)
O1–Pd1–O1^*i*^	180.000 (1)	O1–Pd1–N1^*i*^	93.06 (16)
O1^i^–Pd1–N1^*i*^	86.94 (16)	O1^*i*^–Pd1–N1	93.06 (16)
O1–Pd1–N1	86.94 (16)	N1^*i*^–Pd1–N1	180.000

Compound** 2**			

Zn1–N1	2.020 (4)	Zn1–N2	2.027 (4)
Zn1–O1	1.933 (3)	Zn1–O2	1.941 (4)
O2–Zn1–O1	112.32 (16)	O2–Zn1–N2	93.82 (16)
O1–Zn1–N2	114.07 (16)	O2–Zn1–N1	116.48 (17)
O1–Zn1–N1	94.47 (16)	N1–Zn1–N2	126.72 (18)

Symmetry code—*i*: 2 − *x*, 1 − *y*, 2 − *z*.

**Table 3:** Hydrogen bond geometries for **2** (Å, °).

D–H⋯A	*d* (D–H)	*d* (H⋯A)	*d* (D⋯A)	∠D–H⋯A
C14–H14⋯N1	0.98	2.50	2.974 (1)	110
C17–H17⋯N1	0.98	2.38	2.872 (2)	110
C33–H33⋯N2	0.98	2.47	2.935 (2)	109
C36–H36⋯N2	0.98	2.44	2.928 (8)	110

**Table 4:** Antibacterial activities of the ligands and complexes.

Entry	(*μ*g/disc)	Zone of Inhibition (mm)			
		*S. aureus *	*B. cereus *	*Rhizopus *	*E. coli *
**HL1**	20	21	18	0	22
	10	11	8	0	9
	2	0	0	0	0

**1**	20	24	21	0	27
	10	15	11	0	13
	2	4	0	0	6

**HL2**	20	23	22	0	24
	10	10	9	0	11
	2	0	0	0	0

**2**	20	26	27	0	29
	10	14	13	0	16
	2	6	0	0	8

Penicillin	20	36	14	0	0
	10	15	0	0	0
	2	0	0	0	0

DMF	20	—	—	—	—
	10	—	—	—	—
	2	—	—	—	—

**Table 5:** The effect of base and solvent on complex **1** catalyzed Suzuki reaction of 4-bromoanisole with phenylboronic acid.