# Supplementary material for: Pd(II) and Zn(II) Based Complexes with Schiff Base Ligands: Synthesis, Characterization, Luminescence, and Antibacterial and Catalytic Activities
Source: ScientificWorldJournal. 2013 Nov 6;2013:956840. doi: 10.1155/2013/956840 (PMC3836455; doi:10.1155/2013/956840)
Supplement: Supplementary file 1 — Figure: S1 The IR spectra of HL1, 1 and HL2, 2. Figure: S2 Solid-state excitation and emission spectra of HL1 and HL2 at room temperature. [file 956840.f1.doc]

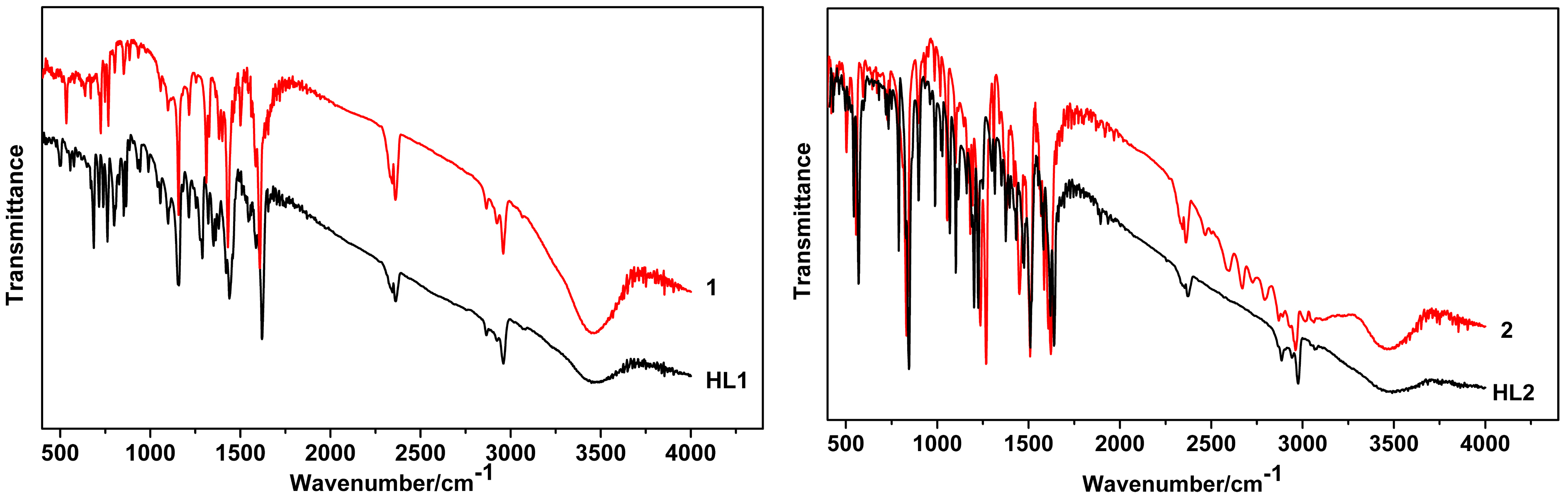


**Fig. S1** The IR spectra of **HL1, 1 and HL2, 2**.


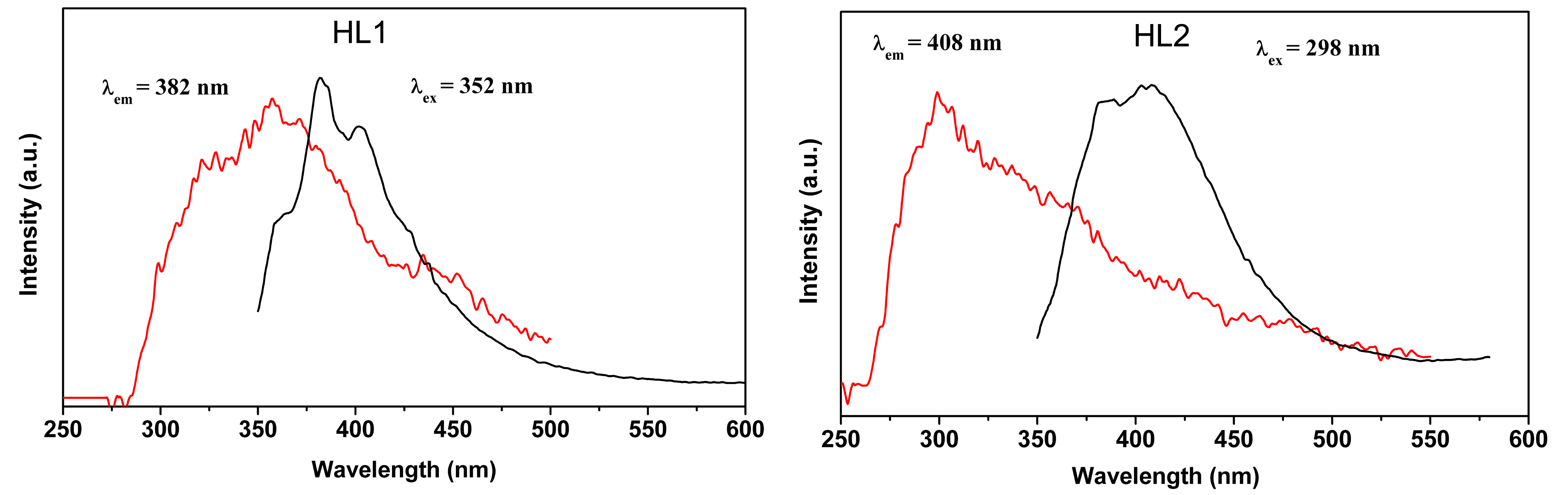


**Fig. S2** Solid-state excitation and emission spectra of **HL1** and **HL2** at room temperature.
